# Supplementary material for: Long-term outcomes of prismatic correction in partially accommodative esotropia
Source: PLoS One. 2019 Dec 2;14(12):e0225654. doi: 10.1371/journal.pone.0225654 (PMC6886759; doi:10.1371/journal.pone.0225654)
Supplement: S1 File — Clinical characteristics of the 124 children with partially accommodative esotropia with a residual esotropia of ≤ 20 prism diopters (PD) after full hypermetropic correction. All children were fitted with prism glasses for more than 1 year and followed-up for 3 years or more unless they underwent strabismus sugery. (PDF) [file pone.0225654.s001.pdf]

SN	onset_age	follow up	SEX	OCCLUSIO	AMBLYOP	Anisomet	DSC_initia	DCC_initia	NSC_initia	NCC_initia	W4D_dist	W4D_nea	PRISM_PI	DCC_PAT	Distance_	Distance_	Distance_	Distance_	NCC_PAT
1	3.9	4.11	1	0	0	0	30	15	40	25	0	0	15	29	.	31	.	.	29
2	3.5	9.82	1	1	0	0	30	10	34	14	0	1	10	12	12	12	26	24	14
3	2.5	9.8	2	1	0	0	30	12	35	17	1	0	12	22	.	.	20	20	22
4	9.2	9.95	2	0	0	0	25	14	25	14	0	0	14	14	12	10	10	8	14
5	8.4	8.4	1	1	1	0	24	12	.	10	1	0	10	12	20	44	30	30	10
6	9.8	9.87	1	0	0	0	20	12	20	12	1	1	12	12	.	12	4	0	12
7	4.0	9.9	2	0	0	0	25	6	25	6	1	0	6	10	.	6	0	6	6
8	2.5	9.87	1	0	0	0	26	10	.	12	1	1	12	18	.	18	20	22	26
9	9.8	9.85	2	1	0	0	22	8	24	10	1	1	8	16	14	14	14	20	10
10	9.6	9.82	1	1	1	1	20	10	30	12	0	0	10	10	.	14	18	16	12
11	4.5	9.52	2	0	0	0	24	12	30	18	1	1	12	24	18	16	16	16	30
12	7.0	8.73	1	0	0	0	33	18	40	16	0	0	18	18	18	18	.	14	16
13	8.0	7.95	2	0	0	0	36	20	.	.	0	0	20	28	28	22	10	4	28
14	7.5	7.63	1	1	1	1	24	12	30	18	1	1	6	12	18	18	18	18	18
15	7.3	7.5	1	0	0	0	24	12	.	0	0	0	15	24	15	15	.	.	20
16	7.3	7.3	2	0	0	0	24	12	30	16	1	1	0	23	15	30	.	.	23
17	7.0	7.23	1	1	0	0	20	12	24	12	0	0	12	24	.	12	12	16	12
18	3.8	7.18	1	0	0	0	30	16	30	16	1	0	16	16	14	6	8	6	16
19	6.0	7.15	1	1	1	1	27	12	.	.	0	0	15	15	7	12	18	20	23
20	4.1	6.75	1	0	0	0	19	10	24	18	0	0	0	12	12	10	10	16	18
21	6.0	6.71	1	1	0	0	20	8	20	8	1	1	14	20	14	13	14	16	15
22	1.5	6.59	2	0	0	0	18	6	20	8	.	1	6	10	12	6	6	7	8
23	5.8	6.5	1	0	0	0	24	12	24	12	1	1	12	22	18	18	22	24	20
24	5.1	6.39	1	1	0	0	16	8	12	4	.	1	8	20	28	16	10	8	10
25	6.0	6.09	2	0	0	0	32	16	32	20	0	0	16	34	28	24	.	30	34
26	4.5	5.82	2	1	0	0	30	15	30	12	0	0	15	24	23	30	.	.	19
27	4.4	5.17	1	1	0	1	36	18	.	0	0	0	18	18	32	34	.	.	0
28	4.9	5.57	1	1	0	0	32	16	32	12	1	1	16	24	16	14	14	18	24
29	3.4	3.5	1	0	0	0	25	12	25	12	0	.	12	24	12	12	4	6	12
30	3.2	5.5	1	0	0	0	25	15	25	10	1	0	15	29	24	29	18	22	33
31	2.5	3.38	1	0	0	0	25	8	25	8	1	1	8	14	16	6	6	8	14
32	5.1	5.38	1	0	0	0	24	10	.	.	1	0	14	20	.	14	10	18	14
33	3.4	5.34	2	1	0	0	16	12	20	12	1	1	12	16	6	6	6	6	12
34	2.1	3.31	2	1	1	0	28	14	28	12	1	1	14	20	16	14	14	10	14
35	5.1	5.26	2	1	0	0	25	14	28	14	1	1	14	22	.	14	14	20	14
36	4.5	5.14	1	0	0	0	25	12	12	0	1	0	12	12	16	16	10	8	20
37	5.1	5.12	1	1	1	0	24	14	24	12	1	1	14	20	14	26	.	.	20
38	5.0	5.12	2	0	0	0	28	14	.	.	1	1	14	22	.	14	6	22	22
39	5.0	5.03	2	1	0	0	24	12	24	12	.	0	12	20	.	12	18	18	12
40	4.3	4.98	1	1	1	0	30	8	25	3	1	1	8	10	8	6	2	4	3
41	4.7	4.9	2	1	0	0	24	8	24	12	1	1	8	20	16	20	20	16	12
42	4.7	4.84	1	1	0	0	40	16	35	11	0	0	16	18	16	14	14	8	24
43	3.0	4.82	2	1	0	0	30	15	30	14	0	0	15	22	15	15	15	24	15
44	4.8	4.8	2	1	0	0	16	10	14	8	.	1	10	10	.	6	0	-4	10
45	4.6	4.76	1	1	0	0	30	14	18	2	1	1	14	14	.	12	6	6	12
46	3.3	4.75	2	1	1	0	40	16	50	20	1	1	16	20	14	14	6	8	20
47	4.1	4.71	1	1	1	0	20	10	.	14	1	1	10	16	10	14	12	12	14
48	4.5	4.59	1	1	0	0	24	12	24	.	0	0	12	26	.	12	12	18	12
49	2.1	4.56	1	1	0	0	32	16	32	.	1	1	16	26	20	22	22	28	30
50	4.3	4.5	1	1	0	0	18	15	18	15	1	1	15	18	.	15	14	8	15
51	4.4	4.46	2	1	1	1	28	19	43	34	0	.	19	28	15	13	11	13	34
52	2.0	4.4	2	1	0	0	35	15	35	15	1	.	15	22	13	11	9	9	15
53	4.0	4.1	1	1	1	1	26	13	26	8	0	0	13	30	.	.	.	20	25
54	2.8	4.1	2	0	0	0	25	15	35	25	1	1	15	18	.	15	10	6	25
55	3.2	4.07	2	0	0	0	15	4	12	1	0	0	4	12	3	4	0	0	1
56	4.0	3.93	1	1	0	0	20	10	24	.	0	0	10	22	10	8	12	14	24
57	3.8	3.86	1	1	0	0	40	12	30	10	1	1	12	24	6	0	0	0	12
58	3.8	3.82	1	0	0	0	16	16	16	16	1	1	16	16	16	16	8	4	16
59	3.0	3.81	2	1	0	0	16	8	24	12	1	1	8	16	.	13	22	15	14
60	3.1	3.79	2	1	1	0	18	14	18	14	1	1	14	20	18	14	8	6	16
61	3.7	3.77	2	1	0	0	28	15	40	27	.	.	15	28	.	15	12	12	27
62	3.5	3.72	1	1	1	0	25	16	28	19	1	1	16	16	10	8	12	16	18.5
63	0.5	3.65	1	1	0	0	25	10	25	12	0	0	10	18	16	16	22	28	12

64	3.1	3.64	2	1	0	0	24	15	30	21	.	0	15	24	.	15	18	19	24
65	3.6	3.58	1	1	1	0	30	15	30	15	0	.	15	18	15	11	3	0	15
66	3.5	3.57	2	0	0	0	40	16	40	16	1	1	16	20	16	20	15	15	22
67	3.4	3.44	2	0	0	0	28	15	28	12	0	0	15	24	20	19	22	24	15
68	3.2	3.4	1	1	1	0	24	15	16	7	.	0	15	15	.	13	18	18	15
69	3.0	3.3	2	1	1	1	24	16	19	11	0	.	16	16	18	18	16	20	16
70	1.8	3.27	2	1	0	0	25	14	25	14	0	.	14	28	8	0	-6	0	14
71	3.0	3.22	1	1	0	1	27	15	24	12	0	0	15	20	13	25	24	.	12
72	2.9	3.19	2	0	0	0	14	8	16	10	1	1	4	12	.	4	4	0	10
73	3.0	4.57	1	1	1	0	24	16	34	12	1	1	16	26	16	12	18	22	26
74	3.0	3.04	1	1	0	0	22	6	16	10	1	1	6	24	22	20	18	22	16
75	2.9	2.94	1	1	1	0	24	15	20	11	0	0	15	24	.	20	20	22	21
76	2.6	2.93	2	0	0	0	30	14	38	22	0	.	14	14	14	14	6	6	21.5
77	2.7	2.74	2	1	0	0	12	15	30	33	0	0	15	22	.	27	.	.	33
78	1.3	2.71	1	0	0	0	30	10	35	6	1	1	10	22	22	20	24	.	10
79	2.3	2.37	1	1	0	0	40	14	33	16	.	.	14	16	.	14	15	10	16
80	1.0	2.35	1	1	0	0	28	15	26	15	.	0	15	24	.	22	22	24	15
81	3.0	2.28	2	1	1	0	24	16	40	16	0	0	16	32	20	18	20	24	32
82	2.2	2.23	1	0	0	0	22	12	25	12	0	0	12	20	24	22	20	20	12
83	1.7	1.75	1	0	0	0	35	14	35	14	.	.	14	14	0	10	10	10	14
84	1.0	2.07	1	1	1	0	28	16	28	16	0	0	16	24	.	16	22	.	16
85	2.0	2.06	2	1	0	0	28	14	38	14	0	0	14	22	.	28	23	30	14
86	1.5	1.66	2	0	0	0	28	14	28	14	.	0	14	20	20	20	20	20	14
87	1.3	1.59	2	1	1	0	28	15	28	15	0	0	15	27	.	27	24	26	17
88	1.5	1.54	2	1	0	0	26	14	33	17	0	0	14	14	14	15	.	.	23
89	0.5	1.48	2	0	0	0	27	15	30	15	0	0	15	15	.	22	27	24	15
90	1.5	1.41	1	0	0	0	22	12	22	12	0	0	12	12	18	18	18	22	24
91	1.2	1.27	2	0	0	0	10	10	10	10	.	0	10	10	6	2	2	0	10
92	1.2	1.47	1	0	0	0	22	6	24	12	0	0	10	10	.	14	22	.	18
93	1.0	1.17	1	0	0	0	28	12	30	18	.	0	14	12	14	14	18	28	18
94	1.0	1.1	1	0	0	0	16	8	28	12	0	0	8	20	8	18	18	12	12
95	1.0	1.05	1	1	0	0	35	15	35	15	.	1	15	15	15	12	16	16	15
96	1.0	1.04	1	1	0	0	30	16	30	15	0	0	15	16	16	22	22	28	15
97	1.0	1.03	2	0	0	0	20	8	60	23	0	0	8	10	.	.	39	.	22.5
98	1.6	0.88	2	1	0	0	27	12	30	10	0	0	10	12	10	18	18	24	10
99	1.0	3.24	2	1	0	0	35	15	43	23	0	0	15	27	.	15	21	.	32
100	1.8	3.46	1	0	0	0	24	12	28	16	0	0	16	30	.	.	.	22	24
101	1.5	2.95	2	1	1	0	27	20	40	30	0	0	15	20	17	28	22	22	30
102	9.9	9.8	2	1	1	0	22	15	30	17	1	0	15	31	23	19	32	.	31
103	7.7	7.89	1	1	1	0	18	8	28	20	1	1	14	14	6	19	.	.	20
104	2.0	9.41	2	1	0	0	20	6	20	6	1	0	13	20	23	14	14	18	21
105	7.4	9.25	1	1	0	0	24	12	16	4	1	1	10	14	.	22	.	.	14
106	7.8	8.26	1	0	0	0	18	13	28	12	1	1	13	13	13	13	20	26	22
107	7.0	7.76	2	0	0	0	16	4	16	8	1	1	8	12	.	14	14	.	12
108	6.1	6.14	2	1	0	0	18	13	24	10	1	1	13	18	14	14	14	12	15
109	3.9	3.96	2	0	0	0	30	10	30	10	0	0	13	18	13	13	.	.	23
110	1.0	5.57	2	0	0	0	40	8	40	8	1	1	8	24	8	.	8	8	8
111	3.0	5.5	2	1	1	0	33	10	35	10	.	.	10	24	.	6	4	10	10
112	5.0	5.3	2	0	0	0	25	8	20	8	1	1	6	14	.	4	4	0	10
113	5.2	5.27	1	0	0	0	50	20	45	20	1	1	20	28	.	12	6	14	20
114	2.5	4.92	1	1	1	0	24	12	24	8	1	1	6	20	.	6	12	24	15
115	1.7	4.67	2	1	0	0	40	14	50	24	.	1	14	14	10	10	.	.	24
116	4.0	4.75	2	1	0	0	24	10	24	8	1	1	10	10	10	10	14	22	16
117	2.3	4.54	1	1	0	0	20	18	28	18	0	0	18	18	.	12	14	20	18
118	2.8	4.07	2	1	1	0	22	10	20	10	1	0	12	12	12	22	.	.	10
119	3.0	3.71	1	1	0	0	27	10	26	16	.	0	15	25	15	15	16	20	31
120	3.3	3.27	1	1	0	0	25	14	30	19	.	.	14	20	14	14	14	10	19
121	0.1	2.9	2	1	0	0	20	8	28	17	.	0	15	19	.	15	12	18	19
122	2.3	2.58	2	1	0	0	25	12	30	17	0	0	12	20	20	16	14	10	17
123	2.9	3.03	2	1	0	0	20	16	33	25	0	.	16	16	16	16	14	8	25
124	1.5	3.44	2	1	0	0	16	14	28	26	0	0	14	24	10	-2	-4	8	25.5

Near_6M	Near_12M	Near_24M	Near_36M	st_0_logm	st_1_logm	st_3_logm	st_6_logm	st_12_logr	st_24_logr	st_36_logr	W4D_dist	W4D_near	Success	Operation	WEANING
.	31	.	.	3.54	3.54	3.54	3.54	3.54	3.54	3.54	.	.	2	1	0
12	12	36	24	3.54	3.54	3.54	3.54	3.54	3.54	3.54	0	0	2	0	0
.	.	20	20	2.3	2.3	2.3	2.3	2.3	1.85	2.6	.	.	2	0	0
14	10	10	8	3.54	3.54	3.54	2	1.85	2	2.15	0	0	1	0	0
16	39	0	0	3.54	3.54	3.54	3.54	3.54	3.54	3.54	0	0	2	0	0
.	12	4	0	3.54	3.54	2	2	1.85	1.7	1.85	1	1	1	0	1
.	6	0	6	3.54	3.54	3.54	3.54	3.54	3.54	2.3	1	0	1	0	0
.	11	12	8	2	1.85	2.15	2.15	1.85	2.6	2	1	1	2	0	0
0	8	2	6	.	.	2	2.3	2	2.15	2	1	0	2	0	0
.	14	4	4	2.3	2.3	2.3	2.3	2	1.85	2	1	1	2	0	0
18	16	18	16	2	1.85	2	2.15	2.3	1.85	2	1	1	2	0	0
15	15	.	14	2.15	2.15	2.15	2.3	2.3	2.3	2.3	0	0	2	0	0
15	16	10	4	.	.	.	.	2.6	2.6	2.6	1	1	1	0	0
6	2	0	0	3.54	3.54	3.54	2	2	1.7	1.7	1	1	2	0	0
15	29	.	.	1.85	1.85	1.85	1.85	1.85	1.85	1.85	.	.	2	1	0
15	30	.	.	.	.	2.15	2	2	2	2	.	.	2	1	0
.	8	6	4	2	2	2	2	1.85	1.85	2	1	0	2	0	0
14	0	4	6	.	.	.	.	2.6	2.6	2.6	0	0	1	0	0
7	1.5	0	0	.	.	.	3.54	3.54	.	.	.	.	2	0	0
20	18	18	20	2.3	2.3	2.3	2.15	2	1.85	2	1	1	2	0	0
24	13	14	20	.	2	2	2.3	2	2.6	2	1	1	2	0	0
12	6	6	7	.	.	.	2	2	2.15	1.7	1	1	1	0	0
8	8	26	12	3.54	3.54	3.54	3.54	1.85	1.85	1.85	1	1	2	0	0
10	10	10	8	3.54	3.54	3.54	3.54	3.54	2.3	2	.	1	1	0	0
26	34	.	30	.	.	.	.	.	.	.	1	0	2	0	0
23	30	.	.	2	1.7	2	2	1.6	1.6	1.6	.	.	2	1	0
32	28	.	.	.	.	.	3.54	3.54	3.54	3.54	.	.	2	1	0
16	14	14	14	2	2	1.6	1.3	2	2	1.6	1	1	2	0	0
12	12	4	6	.	.	.	2.3	2	2.6	2	1	1	1	0	0
15	35	15	12	2.6	2.6	2.6	2.6	2.6	2.3	2.3	.	1	2	0	0
16	6	14	8	2	2	2	2	1.7	1.6	1.85	1	1	1	0	0
.	14	6	8	.	.	.	.	.	2	2	1	1	2	0	0
6	10	10	6	3.54	3.54	2.3	2	2	2	2	.	.	1	0	0
16	14	14	18	3.54	3.54	3.54	2.6	2.6	2.3	1.85	1	1	1	0	0
.	14	14	20	.	1.85	1.85	1.85	2	2	2.15	1	1	2	0	0
18	12	18	12	1.85	1.7	1.85	1.85	1.85	1.85	1.85	1	1	1	0	0
22	30	.	.	.	.	.	2.15	2.15	2.15	2.15	.	.	2	1	0
.	14	6	18	.	.	2.6	2.6	2	1.85	1.85	1	1	2	0	0
.	12	8	8	2.6	2.6	2.6	2.6	2.6	2.3	2.3	1	0	2	0	0
8	6	2	4	.	.	.	1.6	1.85	1.85	1.85	1	1	1	0	0
6	6	2	18	2	2	1.48	1.85	1.48	1.85	1.48	1	1	2	0	0
16	14	14	8	.	.	2.15	1.85	2	1.4	1.85	0	1	1	0	0
15	9	15	11	.	1.85	1.85	2	2	2	1.7	1	1	2	0	0
.	6	0	-4	.	2.6	2.6	2.6	2.6	1.48	2	1	1	1	0	1
.	12	6	6	.	.	.	.	1.85	1.85	1.85	1	1	1	0	0
14	14	6	8	.	.	3.54	2.6	2.6	2.15	1.85	1	1	1	0	0
18	14	12	12	1.85	1.85	1.85	2	1.6	1.85	1.4	1	1	2	0	0
.	12	12	10	.	.	.	.	2	1.85	2	1	0	2	0	0
30	20	14	16	.	2.6	2.6	2.6	2.6	2.6	2.6	1	0	2	0	0
.	15	14	8	2.3	2.3	2.3	2.3	1.85	2	1.6	1	1	1	0	0
15	13	11	13	.	2.6	2.6	2	2	1.6	1.48	1	1	2	0	0
13	11	9	9	.	.	.	2.6	2	2	1.48	1	1	1	0	0
.	.	.	40	.	.	.	.	.	.	.	0	0	2	0	0
.	15	10	6	.	.	.	.	3.54	2	2.15	1	1	1	0	0
3	4	0	0	.	.	.	2.6	2.6	2.6	2.6	0	1	1	0	1
24	22	18	30	2	2	2	2.15	2	1.85	1.7	1	1	2	0	0
6	0	0	0	.	.	.	2.15	1.85	1.85	1.85	1	1	1	0	1
22	20	8	4	2	2	2	1.85	2	2	1.85	1	1	1	0	0
.	13	24	23	1.85	1.85	1.85	1.85	1.85	2	1.78	.	1	2	0	0
20	14	8	6	.	.	.	3.54	1.7	1.7	1.85	1	1	1	0	0
.	15	13	12	.	.	.	.	2	2	2	1	1	2	0	0
10	8	12	16	.	.	.	3.54	3.54	2.6	2	1	1	2	0	0
16	16	16	16	2	2	2	2	2.3	2	2	1	1	2	0	0

.	15	22	24	.	.	.	.	2.3	3.54	2	0	0	2	0	0
15	11	3	0	.	.	.	.	.	2	2	0	0	1	0	1
16	20	15	15	2	2.3	2	2	2	2	2.3	1	1	2	0	0
15	19	22	10	.	2.6	.	1.85	2	1.4	2	0	0	2	0	0
.	7	18	16	.	.	.	.	.	1.85	2.3	1	1	2	0	0
16	4	4	4	.	.	.	.	2.6	2.3	2.3	0	0	2	0	0
8	0	0	0	.	.	.	.	.	2	2.3	0	0	1	0	1
13	25	24	.	.	.	.	2.6	2.6	2.3	2.3	.	.	2	1	0
.	4	4	0	3.54	3.54	2.6	2.6	2	2.3	1.85	1	1	1	0	1
16	16	20	12	.	.	.	2.3	2.6	1.7	1.6	1	1	2	0	0
14	12	8	10	.	3.54	2.3	2.3	2.3	3.54	3.54	1	1	2	0	0
.	7	7	3	.	.	.	.	2.6	2.6	2.6	1	1	2	0	0
16	14	10	6	.	.	.	.	2.15	2	1.6	1	1	1	0	0
.	27	.	.	.	.	.	.	.	.	.	.	.	2	1	0
10	16	24	.	1.85	1.85	1.3	1.85	1.48	2	2	.	.	2	1	0
.	18	15	11	.	.	.	.	3.54	2	2.15	1	1	1	0	0
.	15	13	13	.	.	.	.	3.54	2.6	2.6	.	0	2	0	0
20	18	16	10	.	.	.	.	.	.	3.54	0	0	2	0	0
12	10	16	0	.	.	.	.	.	.	.	0	0	2	0	0
12	10	10	10	.	.	.	.	2	2.6	2.6	1	1	1	0	0
.	8	22	.	.	.	.	.	.	.	.	.	.	2	1	0
.	28	23	30	.	.	.	.	.	2.6	3.54	0	1	2	0	0
26	14	12	14	.	.	.	.	.	2.6	2	1	1	2	0	0
.	15	15	26	.	.	.	.	.	.	.	.	0	2	0	0
36.5	33	.	.	.	.	.	.	2.3	2.3	2.3	.	.	2	1	0
.	15	9	11	.	.	.	.	.	.	3.54	1	1	2	0	0
14	14	12	10	.	.	.	.	.	.	.	1	0	2	0	0
6	2	2	0	.	.	.	.	.	2.3	2.3	.	.	1	0	1
.	14	22	.	.	.	.	.	.	.	.	.	.	2	1	0
18	22	22	24	.	.	.	.	2.3	2.3	2.6	1	1	2	0	0
22	18	20	24	.	.	.	.	.	.	.	.	0	2	0	0
15	12	16	16	.	.	.	.	.	2	2	1	1	2	0	0
25	13	13	14	.	.	.	.	.	.	2.3	1	0	2	0	0
.	17	39	.	.	.	.	.	.	.	.	.	.	2	1	0
10	8	8	16	.	.	.	.	.	.	.	.	.	2	0	0
.	23	22	.	.	.	.	.	2.3	2.6	2.6	.	.	2	1	0
.	.	.	30	.	.	.	.	.	.	2.3	0	0	2	0	0
23	15	18	16	.	.	.	.	2.3	2.3	2.3	1	1	2	0	0
23	25	28	.	.	.	.	2.6	2.6	2.6	2.6	.	.	2	1	0
12	29	.	.	.	.	.	.	3.54	3.54	3.54	.	.	2	1	0
23	18	14	10	2.3	2.3	2.3	2.3	2.3	2	2	1	0	2	0	0
.	22	.	.	2	2	2	2	2	2	2	.	.	2	1	0
13	13	20	26	.	.	.	3.54	3.54	3.54	3.54	1	0	2	0	0
.	14	14	.	1.78	1.85	1.78	1.78	2	1.7	1.7	.	.	2	1	0
13	14	14	12	.	.	.	3.54	2	2	2	1	0	2	0	0
13	25	.	.	.	.	.	.	.	.	.	.	.	2	1	0
8	.	8	8	1.85	2	1.85	1.85	1.85	2	1.7	1	1	1	0	0
.	6	4	10	.	.	.	.	.	.	2.3	1	1	1	0	0
.	4	4	0	3.54	1.85	3.54	3.54	2.15	2	1.85	1	1	1	0	1
.	12	6	14	2.6	2.6	2.6	2.6	2	1.85	1.85	1	1	2	0	0
.	6	12	18	3.54	3.54	2.15	2.15	3.54	2	1.85	1	1	2	0	0
10	10	.	.	.	2.3	.	2.3	2.3	2.3	2.3	.	.	2	1	0
10	18	14	18	3.54	3.54	3.54	2.15	2.15	2	1.85	1	1	2	0	0
.	12	14	20	.	.	.	.	1.85	2.6	2.3	.	0	2	0	0
12	16	.	.	2	1.6	2	2.6	2.6	2.6	2.6	.	.	2	1	0
15	18	18	20	1.85	2.6	1.85	1.85	2.3	2.3	2	1	0	2	0	0
16	16	14	10	.	2.6	2.6	2.6	2.6	2.6	2	.	1	1	0	0
.	18	20	22	.	.	.	.	.	1.85	2	1	1	2	0	0
12	16	14	10	.	.	2.6	2.3	2.3	2	2.3	0	0	1	0	0
16	16	14	8	.	2.6	.	.	1.48	2	1.48	1	1	1	0	0
10	12	0	8	.	.	2	2.15	2	2	2	1	1	1	0	0
